# A prospective cohort study evaluating disease-specific mortality in patients with early-stage Barrett’s esophagus-related neoplasia following endoscopic therapy

**DOI:** 10.1007/s00464-026-12874-7

**Published:** 2026-05-28

**Authors:** Sally Pan, Grace J. Hattersley, Vijayendran Sujendran, Ines Modolell, J. Robert O’Neill, Massimiliano di Pietro

**Affiliations:** 1 Cambridge University Hospitals NHS Foundation Trust, Cambridge, UK; 2 Department of Oncology, Early Cancer Institute, University of Cambridge, Cambridge, UK; 3 School of Clinical Medicine, University of Cambridge, Cambridge, UK; 4 Cambridge Oesophago-Gastric Centre, Cambridge University Hospitals NHS Foundation Trust, Cambridge, UK; 5 Department of Surgery, University of Cambridge, Cambridge, UK; 6 Early Cancer Institute, University of Cambridge, Cambridge Biomedical Campus, Cambridge, CB2 0XZ UK

**Keywords:** Endoscopic eradication therapy, Early esophageal adenocarcinoma, High-grade dysplasia, Endoscopic mucosal resection, Endoscopic submucosal dissection, Barrett’s esophagus

## Abstract

**Background:**

Endoscopic eradication therapy (EET) for Barrett’s esophagus (BE)-related neoplasia is safe and effective in the short term, however there are limited data on long-term outcomes. We aim to provide further evidence on the long-term efficacy of EET in patients with BE-related high-grade dysplasia (HGD) or low-risk T1 esophageal adenocarcinoma (EAC).

**Methods:**

This prospective cohort study enrolled patients with early BE-related neoplasia at a single tertiary referral center between January 2005 and December 2022. We included patients who had a baseline histological diagnosis of HGD, T1a or superficial T1b EAC (invasion into the submucosa less than 500 µ) and received endoscopic therapy with either primary radiofrequency ablation or endoscopic resection (ER). Our primary outcome assessed disease-specific mortality (DSM), which is defined as death following progression to advanced adenocarcinoma not amenable to curative treatment. Secondary outcomes evaluate overall survival, endoscopic recurrence, progression to esophagectomy and major post-procedure complications.

**Results:**

We included 330 patients (HGD *n* = 135; T1a EAC *n* = 170; T1b EAC *n* = 25), of which 283 patients were treated with primary endoscopic resection (ER) whereas 47 patients were treated with RFA alone. Median follow up was 55.35 months (IQR 36.5–86.5). DSM was 2.2% (*n* = 7), with no significant difference between the HGD and EAC groups (*p* = 0.596). Temporal analysis demonstrated outcome improvement, with 85.7% of DSM cases occurring in the 2005–2014 time period. Five-year overall survival was 88.5%. Endoscopic recurrence was found in 4.2% (*n* = 14) of patients and 8.8% (*n* = 29) of patients required surgical intervention.

**Conclusion:**

In BE-related HGD and early-stage EAC, EET was associated with a low rate of progression to surgery and endoscopic recurrence.

**Graphical abstract:**

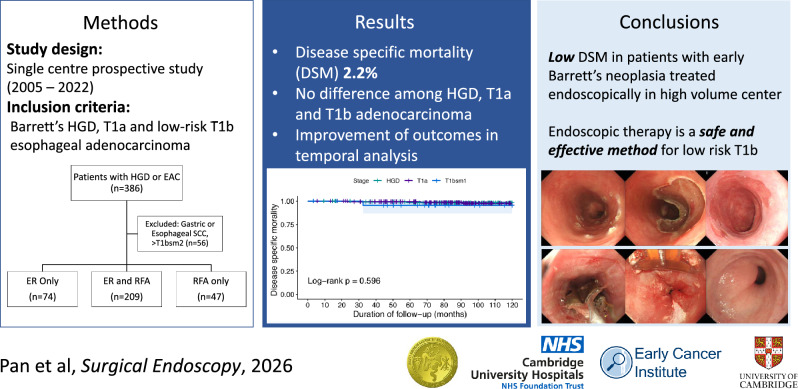

**Supplementary Information:**

The online version contains supplementary material available at 10.1007/s00464-026-12874-7.

Esophageal cancer carries a poor prognosis, with an overall 5-year survival rate of 10–30% [1]. With a sharp rise in incidence of esophageal adenocarcinoma (EAC) globally [2, 3], there has been a significant interest in early detection to allow for minimally invasive treatment of pre-cancerous or early cancer lesions and improve patient outcomes [4, 5].

Barrett’s esophagus (BE) is an intestinal metaplastic change in the distal esophagus resulting from chronic gastroesophageal reflux disease (GERD). BE is associated with a 30-fold increase in the risk of EAC [6, 7]. There is a well described sequence whereby BE progresses to cancer through intermediate dysplastic stages, also known as low-grade dysplasia (LGD) and high-grade dysplasia (HGD). Therefore, endoscopic surveillance of patients affected by BE is recommended by most clinical society guidelines [8–10]. While screening for BE is generally not yet recommended, some guidelines support proactive case finding in people with several risk factors, such as male sex, obesity, age over 50, history of GERD and family history.

The rationale for BE case finding followed by surveillance is that early cancer detection allows curative intervention and less invasive, organ-preserving treatment [10]. However, there has been recent debate about the clinical and cost effectiveness of endoscopic surveillance. A UK randomized controlled study comparing 2-yearly surveillance versus endoscopy-at-need showed no overall and cancer-specific survival advantage associated to regular BE surveillance [11]. Moreover, in this study, 55% of patients diagnosed with cancer during surveillance suffered disease-specific mortality (DSM). This contrasts a longitudinal study published by our group which demonstrated that endoscopic surveillance in an expert center was associated with low DSM [12].

Endoscopic eradication treatment (EET), typically consisting of endoscopic resection, followed by ablation of the residual Barrett’s epithelium, is used with curative intent in HGD or T1 EAC without high-risk features [10, 13]. In cases of HGD without visible lesions or low-grade dysplasia (LGD), ESGE and AGA recommend primary endoscopic ablation therapy [10, 14]. Recent studies have indicated that carefully selected T1b lesions, such as well-differentiated T1bsm1 with no lymphovascular invasion (LVI), may also be amenable to EET instead of surgical management, as recognized in updated guideline recommendations [10]. Furthermore, although esophagectomy remains the mainstay of treatment for T1b EAC with deep submucosal invasion, LVI or poor differentiation [15], recent evidence suggest that the risk of metastasis in patient with complete resection of high-risk T1b EAC is lower than previously thought [16].

However, most studies have focused on short term responses to EET, while long-term outcomes of patients with early BE-related neoplasia have been under-reported in the literature. In some studies, 5-year-survival ranged between 73 and 87.5% [17–19] and progression to surgery was estimated between 0.3 and 10% [17, 20–22]. Rates of recurrence of EAC after 10 years is reported as 4.1% [23] while in an alternative cohort the rate of recurrence of HGD and EAC over a median follow up of 43 months has been reported as 2% [22]. A recent retrospective multi-center study demonstrated the rate of lymph node metastasis in patients with T1a and T1bsm1 undergoing surgery was 5.6% [24].

The present study aims to shed light on the natural history of patients undergoing endoscopic therapy for BE-related neoplasia including HGD and early esophageal adenocarcinoma meeting indication for curative resection. The primary outcome of this study was to evaluate the incidence of DSM in patients with HGD or low-risk T1 EAC undergoing EET. Secondary outcomes included overall survival, endoscopic recurrence, progression to esophagectomy and major post-procedure complications. This cohort study has been reported in line with the STROCSS guidelines [25].

## Materials and methods

The primary source of the data originated from a prospective ethically approved registry study (LREC01/149). Patients providing informed consent did not receive monetary incentive. Clinical and histopathological data from consecutive patients was recorded prospectively in an electronic database between January 2005 and December 2022 at a single tertiary referral center. Any missing data were retrospectively reviewed. Patients received multidisciplinary input and all endoscopic procedures were performed by endoscopists trained in advanced imaging and endoscopic eradication therapy. A small proportion of patients included in this study (< 10%) was not consented to the prospective study. For these patients, data were retrieved via review of electronic healthcare records based on generic consent for research studies. Local governance approval was granted by the institutional review board at Cambridge University Hospitals (QSIS 5105).

Inclusion criteria were: (i) histologic evidence of glandular high-grade dysplasia (HGD) or stage T1 intramucosal adenocarcinoma in the esophagus or at the gastroesophageal junction (GEJ); (ii) at least one session of endoscopic therapy such as endoscopic mucosal resection (EMR), endoscopic submucosal dissection (ESD) or radiofrequency ablation (RFA); (iii) at least 1-year follow up since baseline EET session. We excluded patients with T1b sm2 or sm3 EAC at baseline endoscopic resection (ER) as this is outside the standard curative criteria of EET. Patients with gastric or squamous neoplasia were also excluded.

### Staging and endoscopic management

Patients were assessed for feasibility for EET based on endoscopic assessment by expert endoscopists. Endoscopic ultrasound (EUS) was not routinely performed, but only indicated when expert endoscopic assessment suspected T1b or higher disease or in the follow up or high-risk T1b disease. CT and PET scans were performed selectively at the discretion of the multidisciplinary team (MDT).

Patients with neoplastic visible lesions, who were fit for therapeutic intervention, underwent either EMR or by ESD, which was introduced in our center in 2017. EMR were performed under conscious sedation with Midazolam and Fentanyl with multiband mucosectomy devices (Duette, Cook Medical or Captivator, Boston Scientific). ESD was selected for patients with sessile lesions, early adenocarcinoma larger than 20 mm or residual neoplasia after EMR, which was not amenable to rescue EMR. ESD was performed under general anesthesia as previously described [26]. All patients treated with ER received follow up endoscopy with or without second ER for residual disease at 3 months. Deep margin involvement was not an indication for surgery in the presence of T1a or low-risk T1b disease but triggered vigilant endoscopic follow up with repeat ER in the presence of residual neoplastic lesions and delayed RFA. Residual BE was ablated with RFA, in the appropriate clinical setting, until achievement of endoscopic and histologic remission. Patients with flat dysplasia, and no endoscopically visible lesions were treated with primary RFA. Small Barrett’s islands after at least one RFA procedure were treated with APC. All patients received at least one round of RFA at the GEJ. Patients with disease recurrence or progression under active EET were discussed at MDT to evaluate treatment options that included continued EET or surgery. The indication for an esophagectomy in T1b disease evolved during the study: until 2017 any patient with T1b was considered for surgery, between 2017 and 2021 selected patients with low-risk T1b and good functional status were considered for surgery and from 2022 EET became the treatment of choice of low-risk T1b.

### Outcomes and definitions

The primary outcome of this study was disease-specific mortality (DSM), which is defined as death following progression of disease to EAC stage > 1. Secondary outcomes evaluated endoscopic recurrence, progression to esophagectomy, major post-procedure complications and 5-year overall survival. In patients with HGD on the index procedure, recurrence was defined as HGD or cancer after achievement of complete remission of disease. In patients with EAC, recurrence is defined by histopathological or radiological evidence of EAC after at least two follow up endoscopies negative for cancer. Temporal analysis evaluated disease recurrence, progression to surgery and DSM during two time periods: January 2005–December 2014 (time period 1) and January 2015–December 2022 (time period 2). These two time periods were arbitrarily selected to balance size of the groups and time distribution.

Clinicopathological data including patient sex, age, ASA (American Society of Anesthesiologists) grade, comorbidities, clinical and pathologic stage of early esophageal adenocarcinoma, type of procedure, number of endoscopic procedures, progression to surgery and post-operative complications and survival were obtained from a comprehensive electronic health care record (EPIC, United States).

The extent of BE was measured using the Prague classification and taken prior to the index treatment. Biopsies or ER specimens were reported by at least two expert UGI pathologists according to the modified Vienna classification.

### Statistical analysis

A Kaplan–Meier survival analysis with log rank test was carried out for each primary end point. Continuous variables were summarized by medians and groups compared using the Wilcoxon rank sum test. Categorical variables were compared with Chi-square and Fisher’s exact tests. A *p*-value < 0.05 was considered statistically significant. Analyses were performed using R (v4.5.1; R Core team 2025). There was no prespecified sample size calculation in this study.

## Results

### Study population

**Fig. 1:**
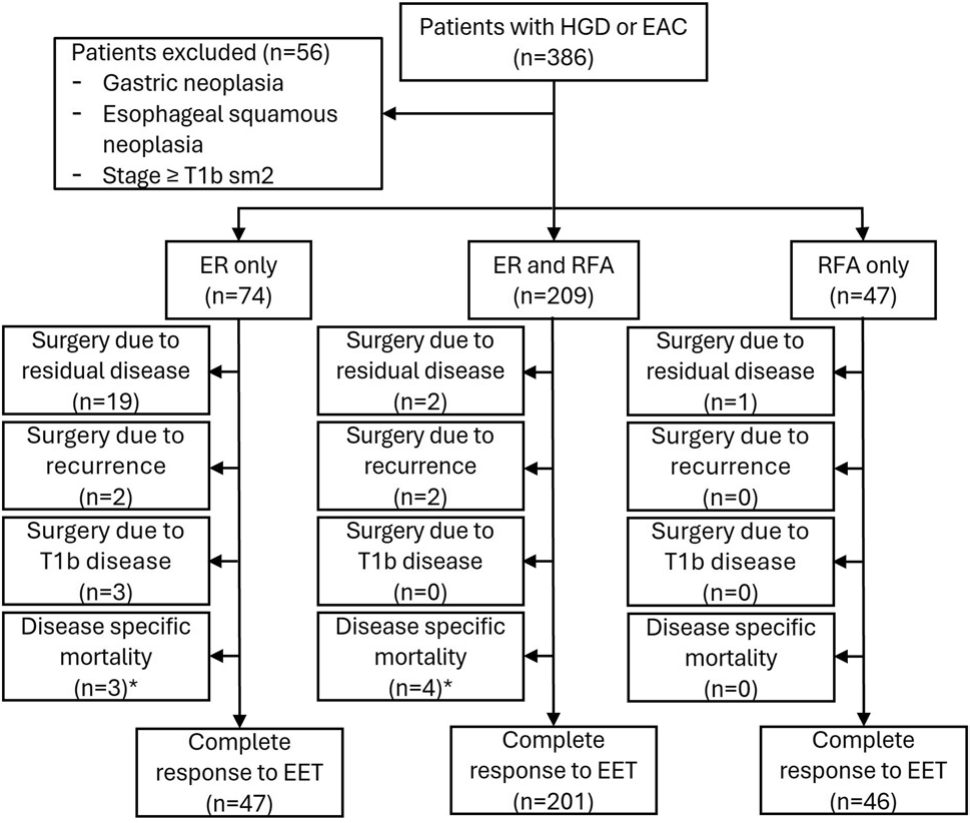
Flowchart summarizing outcomes of patients receiving EET in this study based on the type of endoscopic treatment. Patients in the EMR and RFA group typically received EMR as primary treatment, followed by RFA of residual Barrett’s esophagus. *Patients who have demonstrated significant progression of disease but were unfit or declined surgical intervention

A total of 386 patients underwent EET during the study period and were assessed for inclusion in the study (Fig. 1). Patients who were diagnosed with a baseline histological stage of greater than T1bsm1 or subtypes of gastric and squamous neoplasia were excluded from the study (*n* = 56). Of the 330 patients, 283 patients underwent primary ER (EMR *n* = 275; ESD *n* = 8), which was followed by RFA in 209 patients, whereas 47 patients underwent primary RFA. The characteristics of the study population are detailed in Table 1.

**Table 1:** Cohort demographics

Initial baseline histology	Total cohort (*n* = 330)^†^	HGD (*n* = 135)^†^	EAC (*n* = 195)^†^	*p*-value^‡^
Age at diagnosis	68 (63–75)	67 (60.5–74.5)	69 (64–75.5)	0.15
Gender				
Male	257 (78%)	101 (75%)	156 (80%)	0.33
ASA				0.024
I	148 (45%)	71 (53%)	77 (39%)	0.025
II	145 (44%)	54 (40%)	91 (47%)	0.28
III	32 (10%)	7 (5%)	25 (13%)	0.034
IV	5 (2%)	3 (2%)	2 (1%)	0.4
Proton pump inhibitor use	279 (85%)	115 (85%)	164 (84%)	0.83
Aspirin use	46 (14%)	14 (10%)	32 (16%)	0.17
Prior BE surveillance	218 (66%)	108 (80%)	110 (56%)	< 0.001
Median number of EMR sessions	1 (1–1.75)	1 (0–1)	1 (1–2)	< 0.001
Median number of EET treatments	4 (2–5)	4 (2–5)	3 (2–5)	0.21
Deep margin involvement	21 (6%)	0 (0%)	21 (11%)	< 0.001
Lymphovascular Invasion	18 (5%)	0 (0%)	18 (9%)	< 0.001
Disease recurrence	15 (5%)	6 (4%)	9 (5%)	1
Rate of esophagectomy	29 (9%)	3 (2%)	26 (13%)	< 0.001
Nodal involvement	2 (1%)	0 (0%)	2 (1%)	0.51
Median follow up	55.35 (36.525–86.45)	53.2 (38.4–80.1)	60.4 (35.75–89.9)	0.19

^†^Median (IQR); *n* (%)

^‡^Fisher’s exact test, Chi-squared test

There were no significant differences in terms of age, gender, median follow up time, proton pump inhibitor (PPI) and aspirin use between patients with HGD and early EAC. A greater proportion of patients in the HGD group had pre-existing Barrett’s surveillance compared to the EAC group, reflecting earlier detection with routine surveillance. Patients with EAC (T1a *n* = 170; T1bsm1 *n* = 25) at baseline were more likely to require additional sessions of ER and surgery in comparison to patients with HGD. Moreover, patients with EAC at baseline were more likely to have deep margin involvement and LVI on EET, and nodal involvement if they were to progress to surgery.

### Primary outcome

**Fig. 2:**
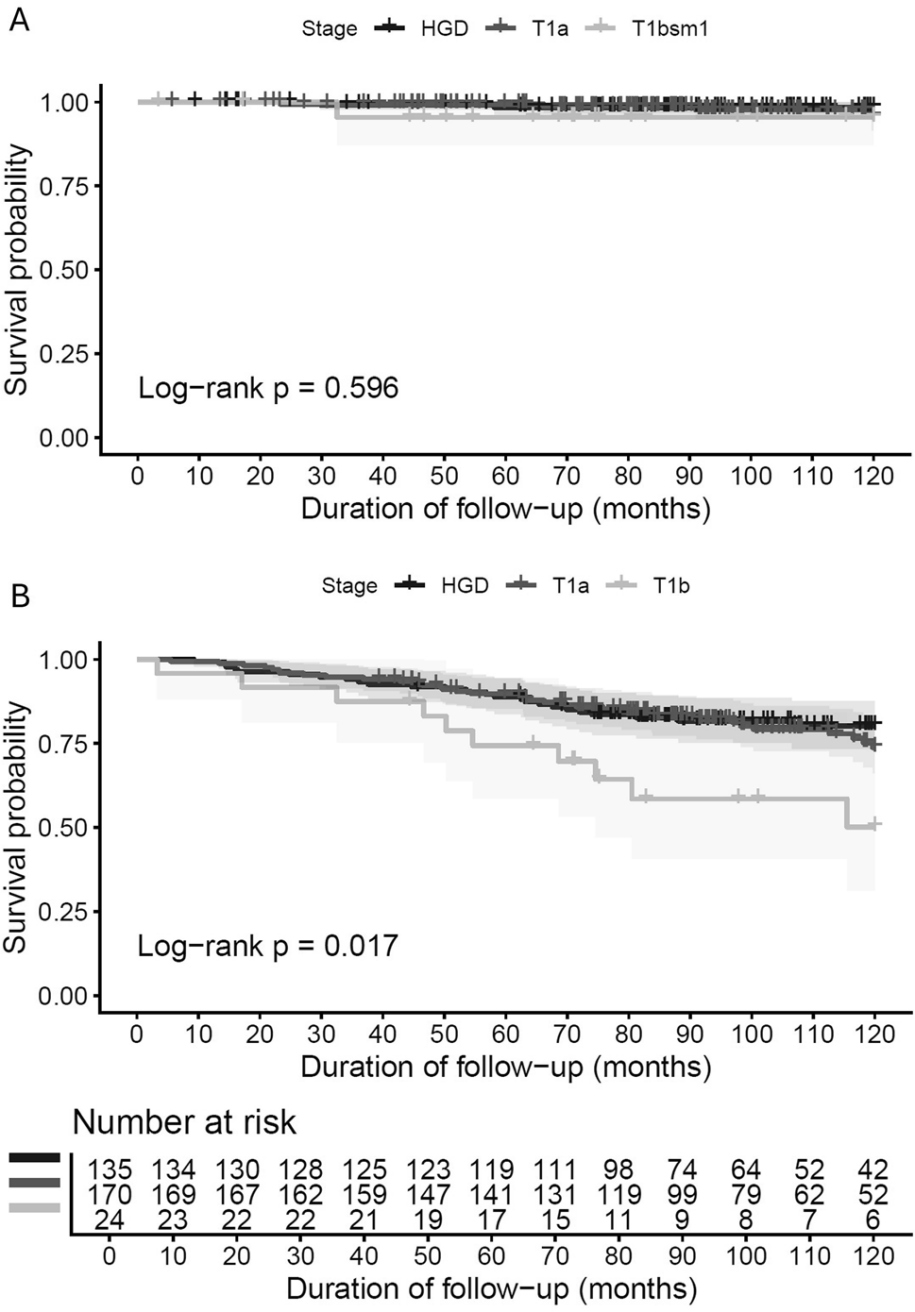
**A** Kaplan–Meier survival curve detailing disease-specific mortality based on baseline histology; **B** Kaplan–Meier survival curve detailing all-cause mortality based on baseline histology

Primary outcome analysis showed that after a median follow up of 55 months, DSM occurred in 2.2% of the patient cohort (*n* = 7, Fig. 2A), with no significant difference found between HGD, T1a, and T1bsm1 groups (*p* = 0.596). Temporal analysis demonstrated that among patients suffering DSM, 85.7% (*n* = 6) underwent their index endoscopic resection during the first period (Table 2).

**Table 2:** Temporal analysis assessing incidence of recurrence, progression to surgery and disease-specific mortality based on time of primary endoscopic resection

Outcome	Total cohort (*n* = 330)	Time period 1 01/2005–12/2014 (*n* = 121)	Time period 2 01/2015–12/2022 (*n* = 209)	*p*-value^‡^
Disease recurrence	15 (4%)	11 (9%)	4 (2%)	0.0044
Progression to surgery	29 (9%)	19 (16%)	10 (5%)	0.0015
Disease-specific mortality	7 (2%)	6 (5%)	1 (0%)	0.011

Fisher’s exact test, Chi-squared test

### Secondary outcomes

The 5-year overall survival in our cohort was 88.5% (*n* = 292). Kaplan–Meier analysis for all-cause mortality (*n* = 82) showed a significant increase in mortality in patients diagnosed with T1b (*p* = 0.017) (Fig. 2B). Within the study cohort, 8.8% of patients (*n* = 29) required surgical intervention due to failure of EET. Among these patients, 75.9% (*n* = 22) was due to residual disease after multiple attempts at EET and 13.8% (*n* = 4) was due to disease recurrence after initial response. During the early stages of the study, prior to 2017, patients with histopathological evidence of low-risk T1b EAC were recommended to undergo surgery. Endoscopic recurrence with a histopathological confirmation was found to be 5% (HGD *n* = 6; EAC *n* = 9), with a median time to recurrence of 34.9 months (IQR 13.02–39.0). Of all endoscopic recurrences, 73.3% (*n* = 11) occurred in patients who underwent index resections in the first time period (Table 2). The overall rate of lymph node metastasis (LNM) was 0% in the HGD and 1.2% (*n* = 2) in the cancer cohort. Among patients who underwent surgery after EET with a baseline diagnosis of EAC, 7.6% (*n* = 2) demonstrated histological evidence of LNM but all patients had clear margins following surgical resection. Among the 10 patients with T1b sm1 who received esophagectomy, 8 had residual early EAC, but none had disease upstage above T1bN0. Major complication rate after ER was 2.4% with four patients experiencing perforation, three patients experiencing a significant bleed and one patient had severe sepsis post procedure, which warranted admission or a secondary procedure.

## Discussion

This study demonstrates that BE-related early EAC, when treated in a high-volume center, carries low DSM with comparable outcome to pre-invasive HGD. We show that EET is associated with good overall survival and a low rate of progression to surgery. Careful patient selection, meticulous operator training and over-arching multidisciplinary team approach have been key to ensure excellent patient outcomes.

When comparing our longitudinal cohort with the available literature, our reported DSM falls within the lower range. A recent international multicenter study found that in T1a EAC, cancer-related mortality was 5% (CI 2–11%; *n* = 106) whereas rate of LNM was 6% (CI 2–12%)[16]. Of the patients who suffered DSM in our study, the majority (85.7%, *n* = 6) underwent their index endoscopic resection treatment between 2005 and 2014. A similar trend was also found with the rate of endoscopic recurrences (73.3%, *n* = 11). There are several reasons that could explain this temporal trend. First, a learning curve could have occurred during the course of the study through refining endoscopic skills, in terms of technique, lesion assessment and risk stratification. Second, technological improvements in the endoscopy system could have impacted on lesion delineation and radicality of the ER. Finally, ESD was introduced in 2017 which could have led to improvement in the management of lesion with submucosal involvement and better staging of lesions.

A recent multicenter study (CONGRESS) assessed the risk of lymph node metastasis (LNM) following endoscopic and/or surgical treatment of T1 EAC and found this to occur overall in 13.5% of cases [24]. Multivariable regression analysis of significant risk factors such as tumor depth, LVI, or signet cells were not found to be significantly associated with LNM or survival but surgery following ER was associated with a significant survival benefit. When directly comparing a selected cohort of patients who progressed to surgery after an endoscopic diagnosis of T1a and T1bsm1 esophageal adenocarcinomas, the LNM rate was 6.9% (*n* = 2) in the present study vs 11.6% (*n* = 18) in the CONGRESS study. We believe that the difference could depend on the careful selection of patients for EET as primary treatment, although it is possible that a proportion of elderly patients who achieved luminal remission could have had subclinical LNM which did not impact on their clinical outcomes.

In our cohort of patients predominantly treated with EMR we found a 4.5% (*n* = 15) rate of endoscopic recurrence. A previous meta-analysis showed that use of EMR is associated with an increase in the recurrence rate of esophageal neoplasia compared to ESD, although this did not hold true for BE-associated neoplastic lesions [26]. Another meta-analysis focused on BE-related neoplasia showed that, compared to EMR, ESD correlated with higher R0 and curative resection rates, but no significant difference was found in the recurrence rate [27]. We have recently reported endoscopic outcomes of EMR and ESD at our institution, which confirmed that the rate of local recurrence in patients treated with en bloc resection was lower than in those treated with piecemeal EMR [28]. Therefore, the predominant use of piecemeal EMR in our cohort could have affected the recurrence rate. However, this rate of recurrence did not jeopardize complete disease eradication as the rate of progression to surgery due recurrence was low (*n* = 4). Our center has recently published the outcomes of 60 consecutive patients referred for esophagogastric lesions, correlating the learning curve with endoscopic outcomes [29]. In that cohort study, 50% of patients had T1b disease reflecting the predominant use of ESD in our center for bulky lesions with suspected submucosal invasion in keeping with ESGE guidelines [10]. Therefore, given that many patients with high-risk T1 disease treated with ESD would have been excluded in the present study, we are unable to compare outcomes of EMR vs ESD.

There is increasing evidence on the efficacy of EET for T1b disease [16]. Our study adds to the growing body of evidence that low-risk T1b should be an absolute indication for EET with comparable outcomes to T1a disease. EET also carries significant lower rate of peri-operative complications and mortality compared to esophagectomy [30]. We excluded high-risk T1b from our study as there is ongoing debate on the efficacy of EET with most guidelines recommending surgery as gold standard treatment. Despite the large, multicenter CONGRESS study [24], there is emerging evidence that the rate of LNM in this group is lower than previously thought. An ongoing prospective study is evaluating the safety and efficacy of EET for high-risk T1b which will provide compelling evidence for future guideline recommendations in treating early EAC, particularly in those with staging greater than T1bsm1, and whether surgical intervention may result in a high survival benefit in comparison to organ-preserving therapy (NCT03222635). Current NICE guidelines could not identify evidence to make concrete recommendations on non-surgical treatment for T1b esophageal adenocarcinoma. Patients with high-risk T1b who are unfit for surgery can be managed with radiotherapy or chemoradiotherapy although the evidence of this topic is scarce [31]. We recognize that our cohort of T1b patients is limited and therefore it is difficult to draw significant conclusions in terms of optimal management of T1b disease. However, our findings are in keeping with current ESGE guidelines, demonstrating that EET offers a safe, organ sparing, curative treatment for low-risk T1b cancers.

There remain some unanswered questions in optimizing the management strategy for patients with early EAC. In our cohort, we utilize long-term follow up strategies but demonstrate a similar, low risk of recurrence when compared to the overall literature. With such low risks of recurrence after successful EET, there may be possibilities to prolong surveillance intervals to decrease the current resource burden. Ongoing developments into optimizing cost-effective screening and surveillance strategies, paired with new endoscopic technologies and biomarker analysis, may guide effective risk stratification and predict recurrence [32].

## Limitations

Our study has several limitations. Although most of the patients were recruited prospectively to an ethically approved registry study, a small proportion of patients included in the present analysis were not recruited to the prospective study. We decided to include these patients as they were managed according to the same clinical standards. In addition, not all the variables were collected prospectively, and retrospective review was required to fill missing data, which could have added some degree of bias. This is a single-center study from a tertiary referral institution; therefore, the results are not directly applicable to units with lower case volume. During the long study period, we note the advancements in technology and degree of endoscopist experience may have evolved. However, strict endoscopy protocols and close multidisciplinary team supervision have been in operation throughout the study. We believe that these aspects are crucial to ensure good patient outcomes. The assessment of DSM is based on putative correlation between death and development of advanced cancer; hence we cannot ensure ascertainment bias. While we might have overestimated DSM, relocation of patients and loss in follow up might have equally led to underestimation. Also, we did not adjust for fitness for surgery, which would have led to underestimation of the rate of progression to surgery and potentially increase rate of DSM if patients could not receive gold standard treatment in case of failure of EET. We were unable to perform multivariable analysis to identify predictors of DSM due to the small number of outcomes. Finally, it is important to consider that our center uses RFA as the primary post EMR ablation technique, therefore the outcome cannot be extended to other ablations modalities such as cryotherapy and APC.

## Conclusion

EET in patients with high-grade dysplasia and T1a esophageal adenocarcinoma was associated with a low disease-specific mortality and low rate of progression to surgery and recurrence. EET provided a safe and effective management strategy for T1bsm1 cancers, but further studies are required to guide optimal treatment of T1b EAC.

## Supplementary Information

Below is the link to the electronic supplementary material.

Supplementary file1 (DOCX 15 KB)

## Data Availability

Datasets generated during study are available on request.

## Declarations

### Artificial intelligence declaration

We have not incorporated AI in the research or development of the manuscript.
