# Supplementary material for: A prospective cohort study evaluating disease-specific mortality in patients with early-stage Barrett’s esophagus-related neoplasia following endoscopic therapy
Source: Surg Endosc. 2026 May 28;40(7):6128–35. doi: 10.1007/s00464-026-12874-7 (PMC13369630; doi:10.1007/s00464-026-12874-7)
Supplement: Supplementary file 1 — Supplementary file1 (DOCX 15 KB) [file 464_2026_12874_MOESM1_ESM.docx]

Supplementary table 1:Subgroup analysis of patients with T1a vs T1bsm1 EAC

| Initial Baseline Histology | | **Total cohort** (n=195) ^†^ | **T1a** (n=170) ^†^ | **T1b** (n=25) ^†^ | **P-value**^‡^ |
| --- | --- | --- | --- | --- | --- |
| **Median number of EMR sessions** | | 1 (1-2) | 1 (1-2) | 1 (1-2) | 0.89 |
| **Median number of EET treatments** | | 3 (2-5) | 4 (2-6) | 2 (1-3) | 0.0018 |
| **Deep margin involvement** | | 21 (11%) | 9 (5%) | 12 (48%) | <0.001 |
| **Lymphovascular Invasion** | | 18 (9%) | 8 (5%) | 10 (40%) | <0.001 |
| **Disease recurrence** | | 9 (5%) | 7 (4%) | 2 (8%) | 0.32 |
| **Rate of esophagectomy** | | 26 (13%) | 16 (9%) | 10 (40%) | <0.001 |
| **Nodal involvement** | | 2 (1%) | 2 (1%) | 0 (0%) | 1 |
|  | ^†^Median(IQR); n(%) | | | | |
|  | ^‡^Fisher's exact test, Chi-squared test | | | | |
